# Rifampicin/Quercetin Nanoemulsions: Co-Encapsulation and In Vitro Biological Assessment Toward Tuberculosis Therapy

**DOI:** 10.3390/ph18121829

**Published:** 2025-12-01

**Authors:** Frank do Carmo Guedes Júnior, Gabriela Hädrich, Camila de Oliveira Vian, Gustavo Richter Vaz, Virginia Campello Yurgel, Daniela Pastorim Vaiss, Gabriela Alves Felício da Costa, Marcelle Oliveira Garcia, Wanessa Maria dos Santos, Beatriz Sodré Matos, Lara Cristina dos Santos Teodoro, João Victor Villa Real, David Nascimento da Silva Teixeira, Alexandre de Paula Rogério, Sergiane Caldas Barbosa, Ednei Gilberto Primel, Pedro Eduardo Almeida da Silva, Daniela Fernandes Ramos, Cristiana Lima Dora

**Affiliations:** 1 Programa de Pós-Gradução em Ciências da Saúde, Laboratório de Nanotecnologia, Faculdade de Medicina, Universidade Federal do Rio Grande, Rio Grande 96203-900, Brazil; frankjrguedes@gmail.com (F.d.C.G.J.); camilavianbio@gmail.com (C.d.O.V.); richtervaz@gmail.com (G.R.V.); virginia.yurgel@gmail.com (V.C.Y.); danipvaiss@gmail.com (D.P.V.); marcelle.garci@gmail.com (M.O.G.); pedrefurg@gmail.com (P.E.A.d.S.); daniferamos@gmail.com (D.F.R.); 2 Department of Pharmaceutical Sciences, University of Vienna, 1090 Vienna, Austria; 3 Escola de Química de Alimentos, Universidade Federal do Rio Grande, Rio Grande 96203-900, Brazil; gabrielaalves753@gmail.com (G.A.F.d.C.); sergianecaldas@furg.br (S.C.B.); eprimelfurg@gmail.com (E.G.P.); 4 Laboratório Experimental de Imunofarmacologia, Universidade Federal do Triângulo Mineiro, Uberaba 38025-180, Brazil; driveuftm@gmail.com (W.M.d.S.); beatrizsodre123@gmail.com (B.S.M.); d202311005@uftm.edu.br (L.C.d.S.T.); joao.villa.real02@gmail.com (J.V.V.R.); david.teixeira@uftm.edu.br (D.N.d.S.T.); alexandre.rogerio@uftm.edu.br (A.d.P.R.)

**Keywords:** nanotechnology, lipophilic drugs, pulmonary diseases, natural products

## Abstract

**Background**: Tuberculosis (TB) remains a leading cause of global mortality, with 1.25 million deaths reported in 2023. Extended treatment duration contributes to poor patient adherence and treatment failure. Innovative drug delivery platforms are needed to improve therapeutic outcomes. **Objective**: This study aimed to develop nanoemulsions co-encapsulating quercetin and rifampicin and evaluate their physicochemical properties and in vitro biological activity relevant to TB therapy. **Methods**: Nanoemulsions (NEs) were prepared via hot solvent diffusion and phase inversion temperature techniques. Physicochemical characterization, stability, anti-inflammatory effects in BEAS-2B cells, and antimycobacterial activity against *Mycobacterium tuberculosis* H37Rv and resistant strains were assessed in vitro. **Results**: The quercetin-rifampicin nanoemulsion (QUE-RIF-NE) showed an average size of 24 nm, zeta potential of −27 mV, and drug recovery rates of 77% (quercetin) and 75% (rifampicin). The formulation was stable and non-cytotoxic at 10^−8^ M, reducing IFN-γ production by half and reactive oxygen species production by almost 75% in BEAS-2B cells. It also exhibited antimycobacterial activity against both susceptible and resistant *M. tuberculosis* strains (MIC ≤ 0.015 µg/mL). **Conclusions**: QUE-RIF-NE exhibits promising physicochemical stability and dual anti-inflammatory and antimicrobial activity in vitro, demonstrating potential for optimized pulmonary or systemic TB therapy that integrates both anti-inflammatory and antimicrobial effects.

## 1. Introduction

Tuberculosis (TB), caused by *Mycobacterium tuberculosis* (MTB), remains a critical global health burden, with 10.8 million new cases and 1.25 million deaths reported in 2023, including 161,000 among HIV-positive individuals. Conventional TB therapy relies on a lengthy six-month oral regimen involving first-line drugs such as rifampicin (RIF), isoniazid, pyrazinamide, and ethambutol. However, severe adverse effects and prolonged treatment duration often undermine patient adherence, contributing to treatment failure and the emergence of multidrug-resistant strains [1,2,3,4].

Rifampicin, a cornerstone of TB treatment, faces formulation challenges due to poor aqueous solubility, limited intestinal permeability, and chemical instability. These factors limit the development of effective liquid formulations and can lead to suboptimal systemic and pulmonary drug concentrations after oral administration. Furthermore, RIF-induced hepatotoxicity is a dangerous side effect that may lead to treatment discontinuation [4,5,6,7,8].

According to previous studies (Hetta et al., 2020) [9], rifampicin-induced hepatotoxicity has been associated with oxidative stress in hepatocytes, resulting from the generation of reactive oxygen species that promote lipid peroxidation and consequently damage the cell membrane. We hypothesize that the co-encapsulation of RIF and QUE into PEG-coated nanoparticles can reduce RIF-induced hepatotoxicity by quenching free radicals and suppressing oxidative stress. Meanwhile, PEGylation enhances biocompatibility and prolongs circulation, thereby minimizing liver toxicity and augmenting hepatoprotective effects through antioxidant and anti-inflammatory pathways [9]. The need to improve rifampicin delivery through novel pharmaceutical formulations and alternative administration routes remains urgent.

Moreover, the use of adjuvant compounds that can enhance the pharmacological effect of rifampicin may also represent an interesting strategy. Quercetin (QUE), a naturally occurring flavonoid, has gained interest as a potential adjuvant compound due to its anti-inflammatory and antioxidant properties. Additionally, QUE may inhibit bacterial efflux pumps and interact with MTB enzymes, such as DNA gyrase and isocitrate lyase, potentially contributing to antimycobacterial activity. Its dual role as an anti-inflammatory agent may also help modulate host responses during TB infection [10,11,12]. In addition, pharmacological interactions between QUE and RIF can enhance RIF’s pharmacokinetic profile, increase its bioavailability while minimizing its side effects, and improve both compounds’ pharmacodynamic characteristics, acting synergistically to promote MTB death [13,14].

However, designing formulations containing lipophilic compounds poses significant difficulties due to their limited aqueous solubility and stability, which negatively impact bioavailability and therapeutic efficacy. Both RIF and QUE, like many anti-TB agents, including the recently approved bedaquiline and pretomanid, are highly lipophilic [15,16]. To address this limitation, lipid-based nanocarrier systems have garnered considerable attention due to their ability to co-encapsulate multiple lipophilic compounds, such as RIF and QUE, thereby improving their solubility, stability, and promoting active and/or passive targeted drug delivery, while also decreasing their drug-induced hepatotoxicity [9,17].

Given the challenges and opportunities, this study aims to develop nanoemulsions (NEs) co-encapsulating RIF and QUE, focusing on their physicochemical characterization and in vitro biological evaluation, including anti-inflammatory and antimycobacterial effects. This approach provides a versatile nanocarrier platform with potential for oral or systemic administration. Here, it is emphasised that a promising nanocarrier formulation is suitable for further development and versatile application routes, including, but not limited to, oral administration, which may ultimately support improved TB therapy outcomes.

## 2. Results

### 2.1. Preparation and Characterization of NEs

The proposed method for the development of the formulations led to the formation of stable NEs capable of encapsulating RIF and QUE efficiently, with the recovery rate of the compounds ranging from 75 to 94%. The NEs had an average particle size ranging from 21 to 43 nm, with a polydispersity index ranging from 0.2 to 0.26, and a zeta potential ranging from −22 mV to −42 mV, as shown in Table 1.

### 2.2. Anti-Inflammatory Activity

#### 2.2.1. Effects of NEs on Cytotoxicity in BEAS−2B Cells

**Figure 1:**
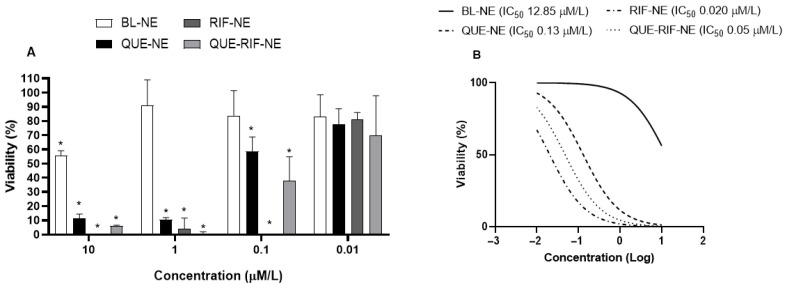
Analysis of the viability of BEAS-2B cells after treatment with BL-NE, QUE-NE, RIF-NE, and QUE-RIF-NE. BEAS-2B cells were treated with different concentrations of BL-NE, QUE-NE, RIF-NE, and QUE-RIF-NE for 24 h—graphs representing % cell viability versus the concentration (**A**) and IC50 values (**B**). The results are expressed as the mean ± SEM of 3 experiments in triplicate. * *p* < 0.05 vs. the negative control (medium).

We observed that QUE-NE, RIF-NE, and QUE-RIF-NE at doses of 10^−8^ M were not cytotoxic to cells, whereas the other doses (10^−6^–10^−8^ M) demonstrated cytotoxic effects, as shown in Figure 1. BL-NE presented a cytotoxic effect only at lower dilutions. BL-NE, RIF-NE, QUE-NE, and QUE-RIF-NE at 10^−8^ M were chosen for the subsequent experiments.

#### 2.2.2. Effects of NEs on Cytokine Productions in BEAS-2B Cells Stimulated by LPS

**Figure 2:**
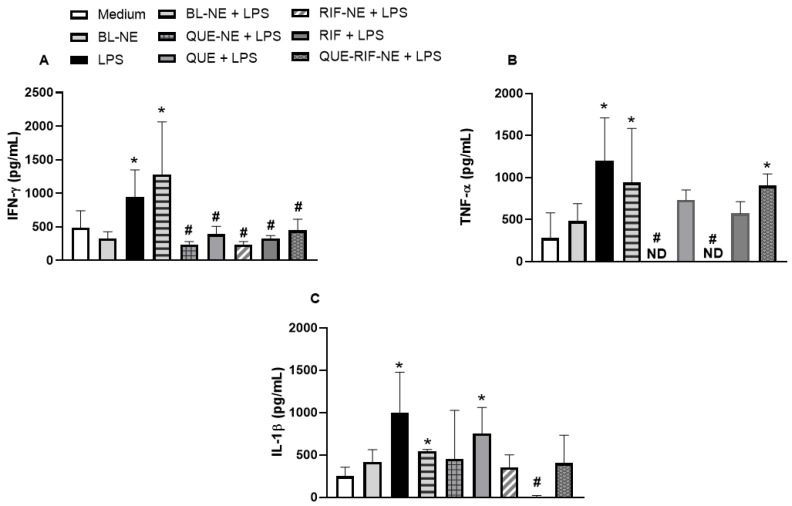
QUE, QUE-NE, RIF, RIF-NE, and QUE-RIF-NE reduced the production of IFN-γ, TNF-α, and IL-1β in bronchial epithelial cells stimulated with LPS. BEAS-2B cells were stimulated with LPS (1 µg/mL) in the presence or absence of BL-NE, QUE, QUE-NE, RIF, RIF-NE, and QUE-RIF-NE (10^−8^ M). After 24 h, the culture supernatants were collected, and the concentrations of IFN-γ (**A**), TNF-α (**B**), and IL-1β (**C**) were measured using an ELISA kit. The data are reported as the means ± S.E.M. (n = 5/group). * *p* < 0.05 vs. the medium, # *p* < 0.05 vs. the LPS. ND = not detected.

Our results demonstrated that LPS increased the production of IFN-γ, TNF-α, and IL-1β compared to the control group (Figure 2A–C, respectively). QUE, QUE-NE, RIF, RIF-NE, and QUE-RIF-NE significantly decreased the IFN-γ production compared to cells stimulated by LPS (Figure 2A). QUE-NE and RIF-NE significantly decreased the TNF-α production compared to cells stimulated by LPS (Figure 2B). RIF significantly decreased the IL-1β production compared to cells stimulated by LPS (Figure 2C). The BL-NE did not cause any significant difference in the cytokine production in cells when compared to the medium group. Cells treated with BL-NE and stimulated with LPS did not present an alteration in the concentration of cytokines when compared to LPS-stimulated cells. In addition, no significant alteration was observed in cells only treated with QUE, QUE-NE, RIF, RIF-NE, or QUE-RIF-NE compared to the negative control (medium).

#### 2.2.3. Effects of NEs on ROS Production in BEAS-2B Cells Stimulated by LPS

**Figure 3:**
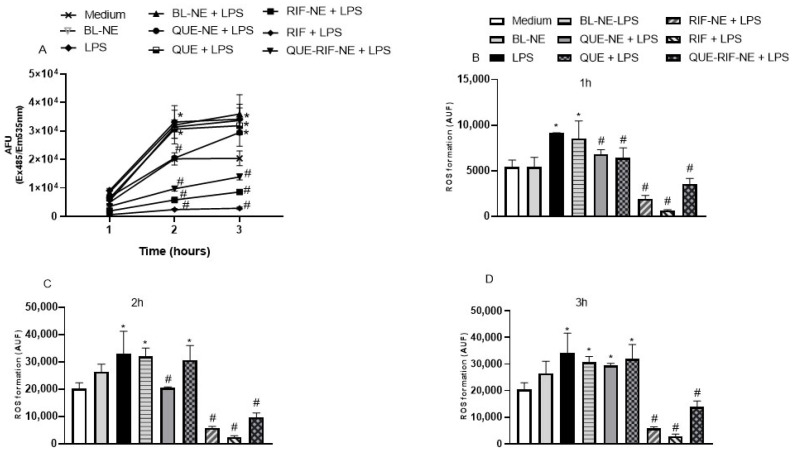
QUE, QUE-NE, RIF, RIF-NE, and QUE-RIF-NE demonstrated antioxidant effects in bronchial epithelial cells stimulated with LPS. BEAS-2B cells were stimulated with LPS (1 µg/mL) in the presence or absence of BL-NE, QUE, QUE-NE, RIF, RIF-NE, and QUE-RIF-NE (10^−8^ M). The fluorescence was measured at an excitation wavelength of 485 nm and an emission wavelength of 535 nm, and expressed in arbitrary fluorescence units (AFU) in kinetics (**A**), 1 h (**B**), 2 h (**C**), and 3 h (**D**). The data are reported as the means ± S.E.M. (n = 5/group). * *p* < 0.05 vs. the medium, # *p* < 0.05 vs. the LPS.

LPS increased ROS production compared to the control group at 1 h, 2 h, and 3 h (Figure 3A–D). QUE, QUE-NE, RIF, RIF-NE, and QUE-RIF-NE significantly decreased the ROS production compared to cells stimulated by LPS (Figure 3A–D) at 1 h after the LPS stimulation, when compared to cells only stimulated by LPS. At 2 h, QUE-NE, RIF, RIF-NE, and QUE-RIF-NE demonstrated antioxidant effects, reducing ROS production. At 3 h, RIF, RIF-NE, and QUE-RIF-NE decreased ROS production. Of note, RIF, RIF-NE, and QUE-RIF-NE reduced the ROS production below basal levels of non-stimulated cells. The BL-NE did not cause any significant difference in the response to produce ROS in cells when compared to the medium group. Additionally, BL-NE did not alter the concentration of ROS compared to cells stimulated by LPS. In addition, no significant alteration was observed in cells treated only with QUE, QUE-NE, RIF, RIF-NE, or QUE-RIF-NE compared to the negative control (medium).

### 2.3. Antimicrobial Activity

A summary of the antimicrobial activity results is shown in Table 2. Free QUE and isolated formulation excipients did not inhibit the growth of any MTB strain at the tested concentrations. NE showed a MIC of 3 µg/mL in the susceptible strain (H37Rv), as free RIF and QUE-RIF-NE showed a MIC ≤ 0.015 µg/mL on the same strain. None of the tested substances demonstrated antimicrobial activity (MIC > 30 µg/mL) against resistant strains with mutation in the *rpoB* gene (RMPR), and only QUE-RIF-NE and Free RIF showed some activity against the MDR strain (FURG-2).

#### 2.3.1. Interaction Between Rifampicin and Efflux Pump Inhibitor

RIF MIC values showed no reduction in the presence of QUE-RIF-NE, Free QUE, and Free RIF (MF < 4) in the FURG-2 strain. However, in the presence of verapamil and chlorpromazine, RIF MIC in FURG-2 returned to the same values obtained in the sensitive strain (MF = 128), showing that there was no efflux pump inhibition activity for the tested NEs. A summary of these results is presented in Table 3.

#### 2.3.2. ROS Production on MTB

**Figure 4:**
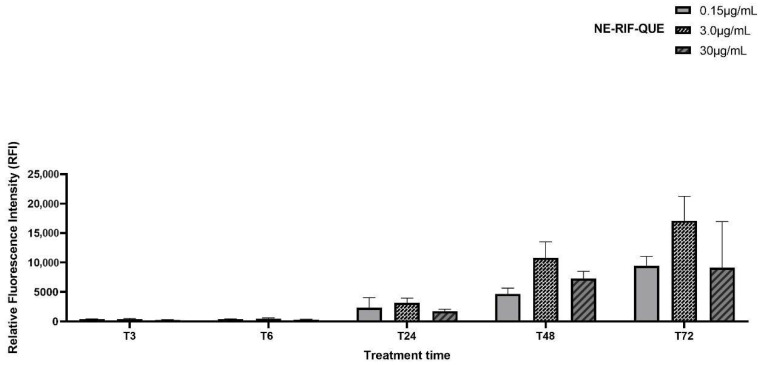
QUE-RIF-NE ROS production on MTB strain at different concentrations at different times (T3 h–T72 h). The results are expressed as the mean ± SEM (n = 3/group).

All tested NE concentrations induced ROS production in the MTB strain over time, with a peak at 72 h; however, a higher, though non-significant, ROS production was observed for QUE–RIF–NE at 3.0 µg/mL (Figure 4).

**Figure 5:**
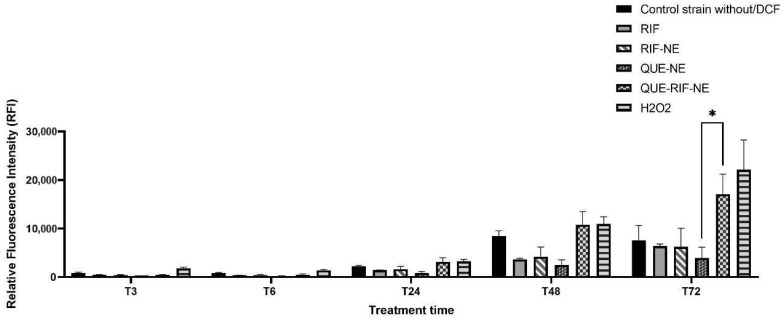
ROS production of different tested compounds on the MTB strain at 3.0 µg/mL concentration at different times (T3–T72 h). The results are expressed as the mean ± SEM (n = 3/group). * *p* < 0.05 QUE-RIF-NE vs. QUE-NE.

No statistically significant difference in ROS production was observed between QUE–RIF–NE and the positive control (H_2_O_2_) at 3, 6, 24, 48, or 72 h (*p* > 0.05). In contrast, ROS production by QUE–RIF–NE was significantly higher than that of QUE–NE at 72 h (*p* < 0.05), highlighting the ROS-generating potential of the co-encapsulated drugs against MTB strains (Figure 5).

## 3. Discussion

Despite the recent progress in TB therapy, effective treatment remains challenging due to several persistent barriers, including poor drug penetration into infected tissues, emergence of multidrug-resistant strains, occurrence of adverse side effects, and long-term duration of conventional therapy. These limitations have intensified the search for innovative therapeutic strategies, particularly those based on nanotechnology, to enhance drug delivery, reduce systemic toxicity, and improve treatment outcomes [18].

Previous studies from our group have already demonstrated the feasibility of encapsulating QUE using a similar nanocarrier system [19,20,21]. However, to the best of our knowledge, this is the first study to report the co-encapsulation of both QUE and RIF for TB therapy. Significantly, in those earlier studies, nanocarrier encapsulation did not compromise the anti-inflammatory activity of QUE, nor did it induce renal or hepatic toxicity—findings that corroborate the safety profile observed in the present work, where no cytotoxicity was detected in bronchial epithelial cells at a concentration of 10^−8^ M (Figure 1).

In vivo studies conducted by Hetta et al. (2020) [9] also demonstrated the potential of nanocarriers for minimizing RIF hepatoxicity. Following intraperitoneal administration of RIF-loaded poly (lactic-co-glycolic acid) (PLGA) nanoparticles into rats, transaminases, oxidative stress markers, and pro-apoptotic genes production were evaluated, showing significantly lower levels of these markers in RIF-loaded nanoparticles-treated groups in comparison to free RIF-treated groups, highlighting the hepatoprotective potential of nanoparticles for RIF use [9].

The study by Hädrich et al. (2016) [22] exemplifies the advantages of quercetin encapsulation in nanosized emulsions, demonstrating significant anti-inflammatory and antioxidant effects with no observed hepatic or systemic toxicity in rats. The potential of nanocarrier systems to enhance bioavailability, reduce oxidative stress (as evidenced by decreased lipid peroxidation), and inhibit pro-inflammatory pathways, such as NF-κB activation, corroborates the hypothesis that the proposed system can mitigate hepatotoxicity [22].

A key outcome of this study was the successful co-encapsulation of QUE and RIF within a nanoemulsion, while preserving their intrinsic pharmacological activities. Lipid-based nanocarriers are widely recognized for their ability to enhance the therapeutic performance of anti-TB agents by improving drug stability, bioavailability, and cellular uptake [23]. The retention of pharmacological activities after encapsulation is particularly significant, as it confirms that the nanocarrier not only acts as a delivery vehicle but also safeguards the antimicrobial, anti-inflammatory, and antioxidant properties of the compounds [24]. This finding is especially relevant in the context of TB therapy, where the harsh pulmonary microenvironment often challenges drug efficacy, the intracellular persistence of MTB, and the requirement for sustained drug release within infected tissues [17].

The present formulation exhibited physicochemical characteristics comparable to those reported in previous studies [25,26], with a monodisperse particle size of approximately 20 nm and a moderate to high negative zeta potential (Table 1). The zeta potential values obtained here are more negative than those reported by Dora et al. (2012) [19] and Hädrich et al. (2015) [22], who observed moderately negative to near-neutral potentials in quercetin-loaded PEG hydroxystearate nanoemulsions. This discrepancy primarily arises from differences in measurement conditions, as well as formulation variables such as surfactant concentration and drug loading (in this case, rifampicin), and batch variation, which affect surface chemistry and charge presentation. PEG-660 stearate contributes to steric stabilization, while the stearate moiety and ionizable drug groups add to the negative zeta potential observed. Overall, the higher negative zeta potentials confirm the stability of nanoemulsions under low ionic strength conditions, complementing previous findings obtained in different measurement environments.

The developed nanoemulsion formulations exhibited superior biological activity compared to the free compounds RIF and QUE. They significantly enhanced anti-inflammatory effects by reducing IFN-γ and TNF-α production (Figure 2). They exerted stronger antioxidant effects by decreasing reactive oxygen species (ROS) generation at both 1 and 2 h (Figure 3). In contrast, free QUE only reduced IFN-γ production and attenuated ROS formation at 1 h. At the same time, RIF alone or combined with QUE demonstrated more pronounced suppression of cytokine release and ROS production (Figure 2 and Figure 3). These results align with previous findings by Gokhale et al. (2019) [27] and Lotfi et al. (2021) [28], who demonstrated that quercetin-loaded nanoemulsions exhibited no cytotoxicity, strongly inhibited LPS-induced TNF-α, and improved the oxidant–antioxidant balance in murine models. Collectively, our data reinforces the potent anti-inflammatory and antioxidant properties of QUE-NE, RIF-NE, and QUE-RIF-NE (Figure 2 and Figure 3), highlighting their potential as promising nanocarriers to enhance therapeutic outcomes in airway diseases [27,28].

Regarding antimicrobial activity, Grotz et al. (2019) reported that polymeric micelles loaded with RIF, with an average particle size of 107 nm, were able to preserve the in vitro antimicrobial activity of the drug, showing MIC values comparable to those of free RIF (7.8–15.6 ng/mL) when assessed using the REMA assay [29]. This observation aligns with the present study, where RIF-loaded NEs demonstrated antimicrobial efficacy comparable to that of free RIF, with no significant difference in MIC values (0.015 µg/mL) against drug-sensitive *M. tuberculosis* strains (Table 2). These findings suggest that nanocarrier encapsulation does not compromise the intrinsic antimicrobial activity of RIF, reinforcing its suitability for incorporation into inhalable delivery systems.

Halicki et al. (2018) [30] also developed RIF-loaded liquid NEs and evaluated their antimicrobial activity against both susceptible MTB H37Rv and MTB MDR strains. Consistent with our findings, their results demonstrated no significant difference in antimicrobial activity between RIF-loaded NEs and free RIF in vitro. Taken together with the data from Grotz et al. (2019), these observations indicate that the encapsulation of RIF within nanocarriers does not compromise its intrinsic antimicrobial activity against strains [29,30].

Galdopórpora et al. (2022) demonstrated that co-encapsulation of RIF with curcumin yielded a formulation suitable for pulmonary delivery, while preserving both the antioxidant activity of curcumin and the antimicrobial efficacy of RIF [24]. In line with these findings, our QUE-RIF-NE also maintained the dual functionality of its active components, retaining the antioxidant potential of QUE and the antimicrobial activity of RIF (Table 2, Figure 3). These parallels reinforce the potential of lipid-based nanocarriers to co-deliver bioactive compounds with complementary pharmacological properties, without compromising their individual activities.

Nevertheless, despite previous reports describing QUE as an efflux pump inhibitor in vitro and in silico against *S. aureus* and carbapenem-resistant Gram-negative bacteria [31,32], such an effect was not observed in the present study. Neither QUE-RIF-NE nor the free drugs were able to reduce the MIC of the FURG-2 strain, which harbors efflux pump mutations. In contrast, classical efflux pump inhibitors significantly decreased the MIC of RIF in the same strain, restoring its susceptibility (Table 3). This discrepancy suggests that the efflux pump inhibitory effects of QUE may be context- or pathogen-dependent, and highlights the need for further investigation to elucidate its potential role as an adjuvant in TB therapy.

Although QUE-RIF-NE did not demonstrate efflux pump inhibition, its ability to enhance ROS generation in MTB strains may still represent an important therapeutic mechanism. In particular, the QUE-RIF-NE induced significantly higher ROS production than QUE-NE and RIF-NE, indicating a synergistic interaction between QUE and RIF that potentiates oxidative stress in MTB strains (Figure 4 and Figure 5). This finding is noteworthy, as increased ROS has been recognized as a secondary antimicrobial pathway in TB treatment, exemplified by drugs such as clofazimine and phenazine derivatives [33].

In addition to enhance ROS production towards MTB, QUE-RIF co-encapsulation may also provide ROS protection to the host cells, like discussed earlier, acting like a oxidative stress producer and defender at the same time, which may improve treatment outcomes due to the complex nature of TB infection, including its ability to produce chronic inflammation on infected tissues and evade host immune cells defenses [19,24,27,28].

The co-encapsulation of QUE and RIF offers a promising dual (antagonistic) mechanism of action through differential modulation of reactive oxygen species (ROS) in mammalian cells and MTB. RIF is well described as inducing oxidative stress in mycobacteria, mediated by elevated levels of superoxide, hydrogen peroxide, and hydroxyl radicals, contributing to its mycobactericidal activity [34]. The described ROS generation in mycobacteria involves the sequential elevation of ROS species, facilitated by NADH oxidases, which enhances the antimicrobial effects of RIF. Conversely, QUE is widely recognized for its antioxidant properties in mammalian systems, effectively scavenging ROS and inhibiting oxidative damage pathways, including NF-κB and Rac1 signaling [35]. Its encapsulation in nanocarriers improves bioavailability and ensures targeted delivery, further enhancing its cytoprotective effects. Notably, QUE mitigates RIF-induced hepatotoxicity by reducing lipid peroxidation and preserving cellular redox homeostasis [22]. This suggests that, depending on the dosage, co-delivery of RIF with QUE nanoparticles can protect host cells from oxidative damage while maintaining or even potentiating ROS-mediated mycobacterial killing.

Here, we hypothesize that QUE’s antioxidant action reduces ROS-induced cytotoxicity in mammalian cells. In contrast, the co-encapsulation of RIF and QUE drives ROS production in mycobacteria, thereby exerting bactericidal effects. The co-encapsulation strategy may thus provide an optimized therapeutic window by enhancing antimicrobial efficacy while simultaneously mitigating host toxicity. Such a dual ROS modulation mechanism aligns with the current understanding of both compounds’ pharmacodynamics and highlights the potential translational advantage of this combination formulation. Thus, rather than compromising activity, the co-encapsulation of QUE and RIF appears to broaden the mechanisms through which the formulation can act against MTB, reinforcing its potential as a multifaceted therapeutic strategy.

Importantly, studies by Marwitz et al. (2024) [36] have demonstrated that pulmonary administration of nanoparticles can enhance drug bioavailability and targeting in the lungs, thereby supporting their potential for effective inhalation therapy. These findings provide a strong foundation for future work, including detailed pharmacokinetic studies and inhalation-specific validation of our system [36].

## 4. Materials and Methods

### 4.1. Reagents

QUE, RIF, verapamil, chlorpromazine, polyethylene glycol stearate (PEG) 660 (Solutol HS15^®^), castor oil (CO), dimethyl sulfoxide 99.5%, resazurin, Middlebrook 7H9 Broth (Difco, Corpus Christi, TX, USA), Middlebrook OADC Enrichment (Difco), were purchased from Sigma–Aldrich (St. Louis, MO, USA). Egg lecithin (LEC) (Phospholipon 80^®^) was purchased from Lipoid (Steinhausen, Switzerland). Methanol was purchased from Pareac (Barcelona, Spain), and distilled water was filtered using a Milli-Q system from Millipore (Burlington, MA, USA). Ethanol, acetone, and other reagents used were of analytical grade.

### 4.2. Preparation of Nanoemulsions (NEs)

**Figure 6:**
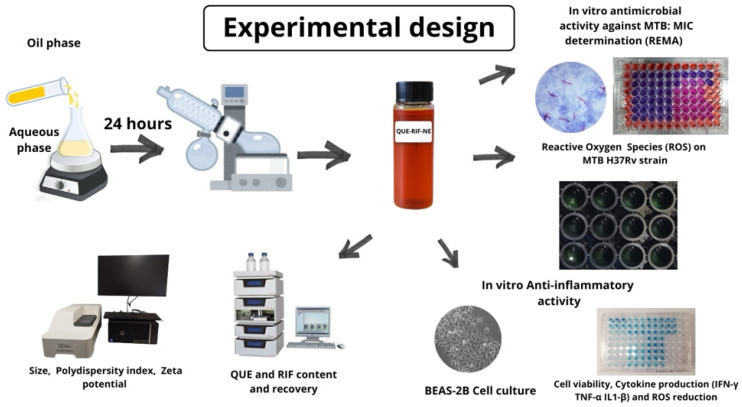
Schematic workflow depicting NEs preparation, characterization, and biological activities evaluation.

A schematic workflow depicting Nes’ preparation, characterization, and biological activity evaluation is shown in Figure 6.

The NEs were prepared using a hot solvent diffusion method in conjunction with the phase inversion temperature technique [22,30]. Briefly, a solution containing either QUE (QUE-NE) or RIF (RIF-NE) or both (QUE-RIF-NE), CO, and LEC was completely dissolved into a mixture of acetone–ethanol (60:40, *v*/*v*) and heated at 60 °C. The resulting organic solution was quickly poured into 50 mL of an aqueous solution of PEG-660 stearate, which had been previously heated to 80 °C and maintained under magnetic stirring at 700 rpm. The resulting colloidal dispersion was then cooled to room temperature for 24 h, the organic solvent was evaporated under reduced pressure, and the final volume was adjusted to 20 mL. Finally, the colloidal dispersion was filtered through a 0.45 μm syringe filter. For the preparation of blank nanoemulsions (BL-NE), neither QUE nor RIF was added to the organic phase of the formulation. Detailed concentrations of compounds used in each formulation are presented in Table 4. All the NEs were prepared in triplicate.

### 4.3. Physicochemical Characterization of the NEs

#### 4.3.1. Size and Polydispersity Index (PDI)

The size and PDI of the NEs were determined by dynamic light scattering (DLS) using an Anton Paar Litesizer™ 500 (Anton Paar GmbH, Graz, Austria). The samples were previously diluted 1000× in ultrapure water. Light scattering measurements were carried out at a 90° angle at a temperature of 25 °C. The hydrodynamic radius was determined using the Stokes-Einstein equation R (kBT/6πD), where kB is the Boltzmann constant, T is the temperature, D is the diffusion constant, and h is the viscosity of the medium.

#### 4.3.2. Determination of Zeta Potential

Zeta potential was determined by electrophoretic light scattering, using an Anton Paar Litesizer™ 500. Measurements were carried out at 25 °C after diluting the samples 1000 times in ultrapure water. The zeta potential values were calculated as mean electrophoretic mobility values using Smoluchowski’s equation.

#### 4.3.3. QUE and RIF Content and Recovery

Liquid Chromatography Tandem Mass Spectrometry (LC-MS/MS) was performed on a Waters Alliance 2695 Separation Module HPLC (High Performance Liquid Chromatography), equipped with a quaternary pump, an automatic injector, and a thermostat column compartment (Waters, Milford, MA, USA). The chromatographic separation was performed by a Luna HILIC 200 Å (Column 100 × 2 mm, 3 µm) (Phenomenex, Torrance, CA, USA). The mobile phase components were (A) ultra-pure water with 0.05% acetic acid and 5 mM ammonium acetate, and (B) methanol with 0.05% acetic acid. Elution was performed in isocratic mode, with an 85% A and 15% B composition, at a flow rate of 0.25 mL/min. The total runtime was 7 min. Mass spectrometry was performed on a Micromass Quattro Micro API (Waters, USA) with an electrospray source. At least two ions (*m*/*z*: parent ion > product ion) were selected for selective reaction monitoring. RIF (823.46 > 791.49 and 823.39 > 398.9) was analyzed in the negative mode, and QUE (301 > 151 and 301 > 179) in the positive mode.

For quantification of samples, external calibration curves were prepared in methanol from 0.01 to 1 mg L^−1^. Samples were diluted in methanol before the analysis.

The QUE and RIF content (total concentration) in the nanocarrier was calculated after determining the drug concentration in the methanolic solutions and was expressed in μg of QUE-RIF/mL. The QUE-RIF recovery was calculated as the percentage of the total drug concentration found in the nanocarrier in relation to the initial added amount.

### 4.4. Anti-Inflammatory Activity

#### 4.4.1. Stimulus and Treatment

The human bronchial epithelial cell line BEAS-2B (ATCC, Manassas, VA, USA) was cultured in DMEM/F12 culture medium according to the manufacturer’s specifications. Cells were incubated (1 × 10^5^ cells/well) in 96-well plates [37] and treated with nanoemulsion (BL-NE; diluted as QUE-NE), QUE, QUE-NE, RIF, RIF-NE, and QUE-RIF-NE at doses of 10^−5^–10^−8^ M for 30 min before stimulation with LPS (1 μg/mL; InvivoGen, Toulouse, France).

#### 4.4.2. Cytotoxicity Assays

The resazurin assay [38] has been utilized to evaluate the effects of substances and nanoemulsions on BEAS-2B cells. Twenty-four hours after treatment, a resazurin solution (2.5 mg/mL, ThermoFisher, EUA, Waltham, MA, USA) was added to each well at 37 °C for 3 h. Fluorescence at 530/590 nm (excitation/emission) was measured on a spectrophotometer (Biotek ION, Agilent, EUA, Santa Clara, CA, USA). Results are presented as the percentage of viable cells relative to the negative control (medium).

#### 4.4.3. Cytokines Measurement

The supernatant was collected 24 h after lipopolysaccharide (LPS) stimulation, and the concentrations of IFN-γ, TNF-α, and IL-1β were measured using enzyme-linked immunosorbent assays (ELISA) according to the manufacturer’s instructions (BD Pharmingen, San Diego, CA, USA).

#### 4.4.4. Test of Reactive Oxygen Species

The kinetics of ROS production were investigated by fluorescence intensity in a microplate assay [39]. Briefly, BEAS-2B cells were stained with 2′,7′-dichlorodihydrofluorescein diacetate (DCFH-DA, 0.1 μM, ThermoFisher, EUA) for 45 min. The supernatant was discarded, and the cells were washed three times with serum-free Dulbecco’s Modified Eagle Medium: Nutrient Mixture F12 (DMEM F12; ThermoFisher, EUA). Subsequently, the cells were treated with substances or nanoemulsions, and after 30 min, stimulated by LPS. Using the EnSpire (multilabel plate reader, PerkinElmer, Rodgau, Germany), fluorescence readings were taken hourly for 3 h to assess the kinetics. Fluorescence was measured at an excitation wavelength of 485 nm and an emission wavelength of 535 nm, and expressed in arbitrary fluorescence units (AFU).

### 4.5. Antimicrobial Activity Assessment in Mycobacterium Tuberculosis Culture

#### 4.5.1. Mycobacterium Tuberculosis Strain Culture

Antimycobacterial activity of compounds was evaluated against MTB RIF-sensitive strain (H37Rv-ATCC 27294), RIF-mono resistant strain (RMPR-ATCC 35838), and MTB multidrug resistant (MDR) strain with mutations in *katG* (S315T) and *rpoB* (S450L) genes (FURG-2) (Table 5). The mycobacteria were obtained from the strain bank of the Medical Microbiology Research Center (Federal University of Rio Grande, Rio Grande, Brazil) and were cultured in Ogawa-Kudoh medium for up to 14 days at 37 °C. From colonies grown in Ogawa-Kudoh medium, bacterial suspensions were prepared in tubes with sterile water containing glass beads. The suspension was homogenized by shaking using a vortex, and the turbidity was adjusted to level 1 of the McFarland scale (3 × 10^8^ CFU/mL). The bacterial inoculum was prepared by diluting the suspension 1:20 in Middlebrook 7H9 medium.

#### 4.5.2. Resazurin Microtiter Assay (REMA)

In a 96-well microplate, a serial microdilution (1:2) of 100 µL of free active ingredients (QUE and RIF) diluted in methanol, BL-NE, QUE-RIF-NE, and the NE excipients was carried out separately in 100 µL of 7H9 medium enriched with 10% OADC (Oleic Acid Albumin Dextrose Catalase). At the end of the microdilution, 100 µL of the standardized inoculum was added. Sterility (negative) and growth (positive) controls were added to each plate. The concentrations evaluated ranged from 30 to 0.015 µg/mL for all tested compounds. QUE-RIF-NE evaluated concentrations stood for RIF concentration.

After seven days of incubation at 37 °C, 30 µL of 0.02% resazurin was added, and the reading was carried out after 48 h of incubation. The minimum inhibitory concentration (MIC) was considered the lowest concentration of the tested compound capable of inhibiting bacterial growth [42]. The color change of resazurin from blue to pink indicates bacterial growth, so the MIC was defined as the absence of color change.

#### 4.5.3. Interaction Between Rifampicin and Efflux Pump Inhibitor

The potential effect of QUE and classic inhibitors (verapamil, chlorpromazine) as efflux pump inhibitors (EPIs) was determined by the Modulation Factor (MF) [43]. The modulation factor reflects the reduction of MIC values of a given RIF concentration in the presence of a subinhibitory concentration of an EPI using the ratio between the MIC of RIF alone and the MIC of RIF in the presence of an EPI. Values of MF ≥ 4 (four-fold reduction) were considered significant.

#### 4.5.4. Reactive Oxygen Species Assay

The ROS measurement was performed according to [44], using the MTB H37Rv strain. The strain was grown in Middlebrook 7H9 medium supplemented with 10% OADC and 0.05% Tween 80 until an optical density (OD_600_) of 0.6–0.8 was reached. The culture was adjusted to an optical density (OD) at 590 nm of 0.5, washed, and stained with 40 μM 2′, 7′-dichlorodihydrofluorescein diacetate (H2DCFDA) for 30 min at 37 °C. In a black solid bottom plate, the H2DCFDA-loaded cells were inoculated in separated wells containing three serial concentrations based on the obtained MIC values (30–3.0–0.15 µg/mL) of QUE-NE, RIF-NE and QUE-RIF-NE, and the ROS production was measured using Tecan Infinite F200 Fluorescence Microplate Reader at the times, in hours, T3, T6, T24, T48 and T72 (510 nm to 535 nm). Hydrogen peroxide (H_2_O_2_) was used as a positive control for oxidative stress, inducing ROS production, and free RIF was used as a positive control for antimicrobial activity, both at the same aforementioned NE concentrations. Intrinsic ROS production of H37Rv strains stained with H2DCFDA (control strain w/DCF), intrinsic strain fluorescence (control strain without/DCF), and intrinsic medium fluorescence (control medium) were also evaluated as controls.

### 4.6. Statistical Analysis

All experiments were performed in triplicate, and average values were used to express the results. Before statistical analysis, data distribution was assessed using the Shapiro-Wilk normality test, confirming that all datasets met the normality assumption. Statistical analysis was conducted using two-way ANOVA followed by Dunnett’s multiple comparisons test. Values of * *p* < 0.05 were considered statistically significant. Graphs were generated using GraphPad Prism version 9.00 (Activation ID: 7c4c55fb-e591–42b6-9167-848618250650).

## 5. Conclusions

The QUE-RIF-NE nanoemulsion developed here successfully preserved rifampicin’s antimicrobial activity and enhanced quercetin’s anti-inflammatory effects, exhibiting low cytotoxicity at low concentrations. Its physicochemical properties suggest a versatile formulation platform suitable for multiple administration routes, beyond a single delivery method. Although quercetin did not inhibit efflux pumps under the tested conditions, its modulation of reactive oxygen species may contribute to an additional antimicrobial mechanism.

By combining pathogen-directed and host-directed actions in a stable nanocarrier, this dual-drug system offers a promising new strategy to improve TB treatment efficacy. Further exploration in advanced in vivo models will be critical to optimizing administration routes and confirming its therapeutic potential.

## Figures and Tables

**Table 1:** Particle size, PDI, Zeta potential, and drug content and recovery.

Formulation	Particle Size (nm ± SD)	PDI (% ± SD)	Zeta Potential (mV ± SD)	Drug Content (µg/mL ± SD)	Recovery (% ± SD)
BL-NE	26.36 ± 8.96	0.22 ± 0.05	−42.26 ± 9.0	-	-
QUE-NE	27.37 ± 2.37	0.26 ± 0.01	−30.73 ± 3.6	1420 ± 60	94.6 ± 4
RIF-NE	21.3 ± 0.94	0.2 ± 0.02	−22.87± 7.3	1200 ± 60	80 ± 4
QUE-RIF-NE	23.72 ± 3.6	0.23 ± 0.3	−26.8 ± 6.7	QUE 578 ± 13.9 RIF 563 ± 5.2	QUE 77 ± 1.85 RIF 75 ± 0.69

NE: Blank Nanoemulsion; QUE: Quercetin; RIF: Rifampicin; QUE-RIF-NE: Nanoemulsion containing QUE and RIF. Results are expressed as the mean and standard deviation (SD) (n = 3/group).

**Table 2:** Minimum inhibitory concentration of tested substances.

Tested Substances	H37RV	RMPR	FURG-2
QUE-RIF-NE	≤0.015 µg/mL (RIF)	>30 µg/mL	0.5 µg/mL
BL-NE	≤3 µg/mL	>30 µg/mL	>30 µg/mL
Free RIF	≤0.015 µg/mL	>30 µg/mL	128 µg/mL
Free QUE	>30 µg/mL	>30 µg/mL	>30 µg/mL
NE excipients	>30 µg/mL	>30 µg/mL	>30 µg/mL

QUE-RIF-NE: Nanoemulsions containing QUE and RIF, BL-NE: blank nanoemulsion; RIF: Rifampicin; QUE: Quercetin.

**Table 3:** Efflux pump inhibitors (EPIs) in combination with RIF against MTB strains.

EPIs	H37Rv		FURG-2	
	RIF MIC	MF	RIF MIC	MF
Verapamil (128 µg/mL)	≤1 µg/mL	0.015	≤1 µg/mL	128
Chlorpromazine (5 µg/mL for sensible strain; and 15 µg/mL for MDR)	≤1 µg/mL	0.015	≤1 µg/mL	128
QUE (0.25 µg/mL)	≤1 µg/mL	0.015	256 µg/mL	0.5
QUE-RIF-NE (RIF 563 µg/mL and QUE 578 µg/mL)	≤1 µg/mL	0.015	128 µg/mL	1.0
Free RIF	≤1 µg/mL	0.015	128 µg/mL	1.0

EPIs: Efflux Pump Inhibitors; MF: Modulation Factor; MIC: Minimum Inhibitory Concentration; RIF: Rifampicin.

**Table 4:** Composition of BL, QUE, RIF, and QUE-RIF nanoemulsions.

Formulation	PEG 660-Stearate (% p/v)	CO (mg)	LEC (mg)	QUE (mg)	RIF (mg)
BL-NE	1.5	150	20	-	-
QUE-NE	1.5	150	20	30	-
RIF-NE	1.5	150	20	-	30
QUE-RIF-NE	1.5	150	20	15	15

PEG: polyethylene glycol stearate; CO: Castor oil; LEC: Egg lecithin; QUE: Quercetin; RIF: Rifampicin; BL-NE: blank nanoemulsions (no drugs); QU-NE: Nanoemulsion containing QUE; RIF-NE: Nanoemulsion containing RIF; QUE-RIF-NE: Nanoemulsion containing QUE and RIF.

**Table 5:** Susceptibility profile and genotypic characterization of MTB.

Strain	Phenotype	*katG*	*inhA* Prom	*rpoB*	*rrs*	*gyrA*	Efflux Pumps
H37Rv-ATCC 27294	Susceptible	Wild Type	Wild Type	Wild Type	Wild Type	Wild Type	-
RMPR-ATCC 35838	Mono-resistant to RIF	Wild Type	Wild Type	H526Y	Wild Type	Wild Type	-
FURG-2	MDR	S315T (AGC🡪ACC)	Wild Type	S450L (TCG🡪TTG)	Wild Type	Wild Type	Increase

MDR: multidrug resistant (*M. tuberculosis* resistant at least to isoniazid and rifampicin); *katG*: gene related to resistance to isoniazid; *inhA* prom: promoter region of *inhA* gene, related to resistance to isoniazid; *rpoB*: gene related to resistance to rifampicin; *rrs*: gene related to resistance to amikacin; *gyrA*: gene related to resistance to ofloxacin; Efflux pump: Overexpression of efflux-related genes—mmpL7, mmr, Rv1258c, p55, Rv2469, efpA, and the transcriptional regulator whiB7—according to [40,41].

## Data Availability

The original contributions presented in this study are included in the article. Further inquiries can be directed to the corresponding author.

## Abbreviations

The following abbreviations are used in this manuscript:

BL-NE	Blank Nanoemulsion
CO	Castor oil
DLS	Dynamic Light Scattering
DNA	Desoxyribonucleic acid
ED	Emitted Dose
EPI	Efflux Pump Inhibitors
FPD	Fine Particle Dose
FPF	Fine Particle Fraction
GSD	Geometric Standard Deviation
HIV	Human Immunodeficiency Virus
HPLC	High-Performance Liquid Chromatography
IFN-γ	Interferon gamma
IL-1β	Interleukin-1 Beta
LC-MS/MS	Liquid Chromatography Tandem Mass Spectrometry
LEC	Egg lecithin
LPS	Lipopolysaccharide
MDR	Multidrug resistant
MF	Modulation Factor
MIC	Minimum Inhibitory Concentration
MMAD	Mass Median Aerodynamic Diameter
MTB	Mycobacterium tuberculosis
NE	Nanoemulsion
NGI	Next-Generation Impactor
PDI	Polydispersity index
PEG	Polyethylene glycol stearate
QUE	Quercetin
QUE-NE	Quercetin Nanoemulsion
QUE-RIF-NE	Quercetin–Rifampicin Nanoemulsion
REMA	Ressazurin Microtiter Assay
RIF	Rifampicin
RIF-NE	Rifampicin Nanoemulsion
RNA	Ribonucleic acid
ROS	Reactive Oxygen Species
TB	Tuberculosis
TNF-α	Tumoral Necrosis Factor Alpha
